# Elevated international normalized ratio contributes to poor prognosis in patients with traumatic lung injury

**DOI:** 10.3389/fmed.2024.1426999

**Published:** 2024-08-02

**Authors:** Qingwei Lin, Enlan Peng, Xingping Deng, Xiaomin Song, Lincui Zhong, Longping He, Qingbo Zeng, Jingchun Song

**Affiliations:** ^1^Intensive Care Unit, The 908th Hospital of Chinese PLA Logistic Support Force, Nanchang, China; ^2^Intensive Care Unit, Changcheng Hospital Affiliated to Nanchang University, Nanchang, China; ^3^Intensive Care Unit, Nanchang Hongdu Traditional Chinese Medicine Hospital, Nanchang, China

**Keywords:** trauma, lung injury, coagulopathy, international normalized ratio, prognosis

## Abstract

**Objective:**

To investigate the pivotal determinants contributing to the adverse prognosis in patients afflicted with traumatic lung injury (TLI), with an aim to mitigate the elevated mortality rate associated with this condition.

**Methods:**

A retrospective analysis was carried out on 106 TLI patients who were admitted to the intensive care unit of a comprehensive hospital from March 2018 to November 2022. The patients were categorized into two groups based on their 28-day outcome: the survival group (*n* = 88) and the death group (*n* = 18). Random forest model, least absolute shrinkage and selection operator (LASSO) regression and support vector machine recursive feature elimination (SVM-RFE) were utilized to pinpoint the primary factors linked to poor prognosis in TLI patients. The Receiver Operating Characteristic (ROC) curve analysis was utilized to ascertain the predictive value of INR in forecasting the prognosis of TLI patients. Based on the cut-off value of INR, patients were categorized into two groups: INR ≥ 1.36 group (*n* = 35) and INR < 1.36 group (*n* = 71). The 28-day survival rate was then compared using Kaplan–Meier analysis.

**Results:**

Random forest model, LASSO, and SVM-RFE jointly identified International standardization ratio (INR) as a risk factor for TLI patients. The area under the ROC curve for INR in predicting the 28-day mortality of TLI patients was 0.826 (95% CI 0.733–0.938), with a cut-off value of 1.36. The 28-day mortality risk for TLI patients with an INR ≥ 1.36 was 8.5 times higher than those with an INR < 1.36.

**Conclusion:**

Traumatic lung injury patients with elevated INR have a poor prognosis. An INR of ≥1.36 can be used as an early warning indicator for patients with traumatic lung injury.

## 1 Introduction

Traumatic lung injury (TLI) represents a significant form of pulmonary damage resulting from severe traumatic events, including vehicular collisions, falls from elevation, and severe crush incidents (1). In the context of polytrauma cases, TLI emerges as the predominant injury pattern, constituting approximately 60% of all trauma-related injuries (2). Due to its unique air-containing structure, lung tissue can suffer from pulmonary contusion, bleeding in the airways, tracheal rupture, hemothorax, tension pneumothorax, coagulation disorders, respiratory failure, and even hemorrhagic shock during trauma (3). TLI is complex and often accompanied by multiple injuries, making diagnosis and treatment difficult in emergency conditions (4). According to literature reports, the mortality rate of TLI patients can reach 11–56% (5).

In addition to direct lung tissue damage caused by trauma, trauma-induced inflammatory response and coagulation disorders are also major mechanisms of traumatic lung injury (6). Traumatic inflammatory responses are usually mediated by toll-like receptors (TLRs), followed by the activation of NF-kB, releasing a large amount of inflammatory factors (7). Inflammatory responses can cause polymorphonuclear neutrophils to be recruited to the site of injury, promoting immune thrombosis and further aggravating tissue hypoxia (8). After lung alveolar capillary membrane damage, the permeability of lung tissue increases, which can aggravate pulmonary edema (9).

Although the pathophysiological mechanisms of TLI have been relatively well-understood, there is still a lack of effective indicators for accurately predicting the prognosis of TLI. The lung injury score is related to the occurrence of ARDS, but it is operationally complex and requires blood gas analysis and chest CT before it can be performed (10). Therefore, this study aims to conduct a retrospective analysis of 106 TLI patients treated at a comprehensive hospital to identify better prognostic indicators and guide treatment.

## 2 Objectives and methods

### 2.1 Subjects and grouping

**Figure 1:**
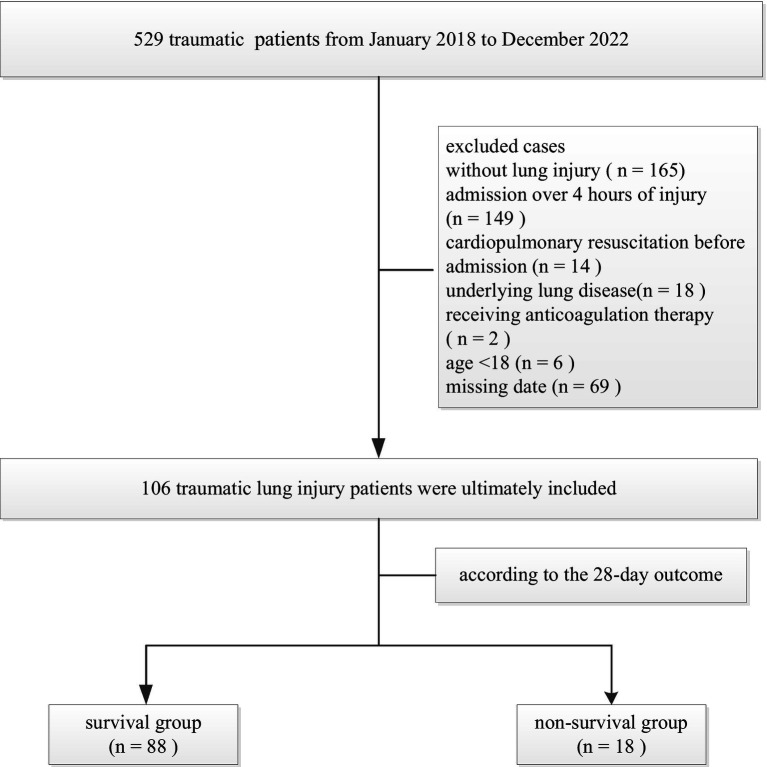
Flow diagram of patient selection and exclusion for traumatic lung injury patients.

A total of 529 traumatic patients admitted to the Intensive Care Unit of the 908th Hospital of Chinese People’s Liberation Army Logistic Support Force from January 2018 to December 2022 were retrospectively analyzed. Inclusion criteria: aged ≥18 years; chest CT results suggest TLI; admission to the intensive care unit within 4 h of injury. Exclusion criteria: without lung injury, known congenital coagulation dysfunction; known underlying lung disease; moderate to severe liver disease; patients receiving anticoagulation therapy; and patients with cardiopulmonary resuscitation before admission. A total of 106 TLI patients were ultimately included (Figure 1). There were 78 males and 28 females, aged 18–88 (48.6 ± 17.5) years. According to the 28-day outcome of TLI patients, they were divided into survival group (*n* = 88) and death group (*n* = 18). This study was approved by the Ethics Committee of the 908th Hospital of the Chinese PLA Logistic Support Force (LC2018028).

### 2.2 Data collection

All patients’ basic data at admission were collected, including gender, age, injury types and sites, Injury Severity Score (ISS), mechanical ventilation and renal replacement treatment. Blood was drawn at the time of admission, and all laboratory analyses were performed within the subsequent 2 h, including blood routine (hemoglobin levels and platelet count), blood gas analysis [oxygenation index (PaO2/FiO2) and serum lactate], routine coagulation tests [activated partial thromboplastin time (APTT), prothrombin time (PT), thrombin time (TT), antithrombin activity, fibrinogen, D-dimer, and fibrin degradation products (FDPs)], and thromboelastography (TEG) [reaction time (R), clot kinetics (K), clot formation dynamics (Angle), maximum Amplitude (MA), and coagulation index (CI)] (11). The length of stay in the ICU and the outcome were also recorded after the patients were discharged.

### 2.3 Statistical analysis

The R 4.3 statistical software was utilized for data analysis, with all analyses conducted on a two-sided basis. Count data were expressed as *n* (%), and compared using the χ^2^ test. For metric data, the single-sample S-W method was used for normality testing. Data that conformed to a normal distribution were expressed as mean ± standard deviation, while non-normal distribution data were expressed as median (interquartile range). When the data were normally distributed and homoscedastic, a *t*-test was used for comparison between groups. When the data were not normally distributed or heteroscedastic, a non-parametric rank sum test (Mann–Whitney U test) was used for comparison between groups.

Three algorithms, LASSO regression, SVM-RFE, and random forest, were employed to identify potential risk factors for outcome in patients with TLI. INR was used to evaluate the 28-day mortality of TLI patients, and the ROC curve analysis was performed. The Kaplan–Meier curve was compared between the INR ≥ 1.36 group and the INR < 1.36 group to assess the 28-day survival rate of TLI patients. *p* < 0.05 was considered statistically significant.

## 3 Results

### 3.1 Demographic data

There was no significant difference in age, gender, injury types, and sites between TLI survivors and non-survivors (*p* > 0.05). However, compared to the survival group, the non-survival group exhibited significantly elevated lactate levels, ISS scores, as well as significantly lower oxygenation indices, hemoglobin concentrations (*p* < 0.05) (Table 1). Simultaneously, the non-survivors with TLI have a higher proportion of ventilator support and continuous renal-replacement compared to the TLI survivors, and they also spend more time receiving treatment in the ICU. Impressively, TLI fatality cases also exhibit more severe coagulopathy characterized by significantly prolonged APTT and PT, increased INR, and decreased platelet counts, fibrinogen, and antithrombin levels (*p* < 0.05). Similarly, the non-survivors with TLI also have significantly abnormal TEG indices, with longer R and K times, lower α, MA and CI values compared to survivors. In contrast, there were no significant difference in D-dimer and FDPs between the two groups (*p* > 0.05).

**Table 1:** Demographic data.

	Survival group (*n* = 88)	Non-survival group (*n* = 18)	*t*/*Z*/χ^2^ value	*p* value
Characteristics				
Age (years)	48.4 ± 16.1	47.0 ± 19.4	−0.332	0.740
Male, *n* (%)	66 (75.0)	12 (66.7)	0.534	0.465
ISS score (IQR)	26 (19, 29)	29 (26, 35)	−2.689	0.007
Injury types, *n* (%)			**0.069**	**0.793**
Blunt injury	80 (90.9)	16 (88.9)		
Sharp injury	8 (9.1)	2 (11.1)		
Other injury sites, *n* (%)				
Head	8 (9.1)	2 (11.1)	0.138	0.711
Abdomen	46 (52.3)	10 (55.6)	0.07	0.791
Pelvis	18 (20.5)	4 (22.2)	0.028	0.867
Limb	50 (56.8)	10 (55.5)	0.010	0.922
Laboratory findings, median (IQR)				
Oxygenation index	281 (220, 360)	138 (63, 218)	−4.390	0.000
Hemoglobin, g/L	100.5 ± 25.7	74.3 ± 26.7	−3.905	0.000
Lactate, mmol/L	2.4 (1.4, 3.7)	5.3 (3.5, 11.2)	−3.624	0.000
Platelet count (×10 ^9^/L)	78 (111, 160)	65 (34, 135)	−2.575	0.01
Fibrinogen (g/L)	1.97 (1.53, 3.61)	1.21 (0.56, 2.98)	−2.394	0.017
APTT(s)	33.6 (28.1, 43.2)	41.6 (36.7, 63.4)	−3.105	0.002
PT(s)	15.2 (13.3, 16.8)	20.9 (16.2, 33.9)	−4.313	0.000
INR	1.24 (1.07, 1.35)	1.70 (1.34, 2.72)	−4.448	0.000
TT(s)	14.1 (11.4, 16.6)	17.4 (13.2, 24.4)	−2.356	0.018
D-Dimer(μg/mL)	7.6 (3.9, 18.8)	11.9 (4.3, 27.0)	−0.850	0.395
FDP(μg/mL)	31.0 (13.3, 67.2)	50.1 (25.9, 93.4)	−1.430	0.153
Antithrombin (%)	64.5 (54.3, 81.8)	54.8 (45.8, 63.0)	−2.579	0.010
TEG parameters				
R(min)	7.2 (5.6, 8.6)	9.6 (7.1, 17.4)	−3.039	0.002
K(min)	2.9 (2.1, 4.2)	4.3 (2.5, 11.3)	−2.269	0.006
Alpha angle (°)	53.9 (41.7, 60.6)	31.8 (17.7, 49.9)	−3.092	0.002
MA (mm)	51.2 ± 12.4	38.9 ± 19.8	−2.516	0.021
CI	−2.8 (−5.9, −1.0)	−6.5 (−12.1, −3.1)	−2.736	0.006
Treatments				
Mechanical ventilation, *n* (%)	53 (66.1)	15 (83.3)	9.698	0.002
Use of continuous renal-replacement, *n* (%)	6 (17)	9 (50)	22.937	0.000
Length of ICU stay, median (IQR), day	13.5 (8, 23)	3.5 (1.0, 18.9)	−3.120	0.002

ISS, Injury severity score; APTT, Activated partial thromboplastin time; PT, Prothrombin time; INR, International normalized ratio; TT, Thrombin time; FDP, Fibrin degradation products; TEG, Thromboelastography; R, Reaction time; K, Kinetics time; MA, Maximum amplitude; CI, Coagulation index.

### 3.2 Screening for predictors of unfavorable outcome in TLI patients

**Figure 2:**
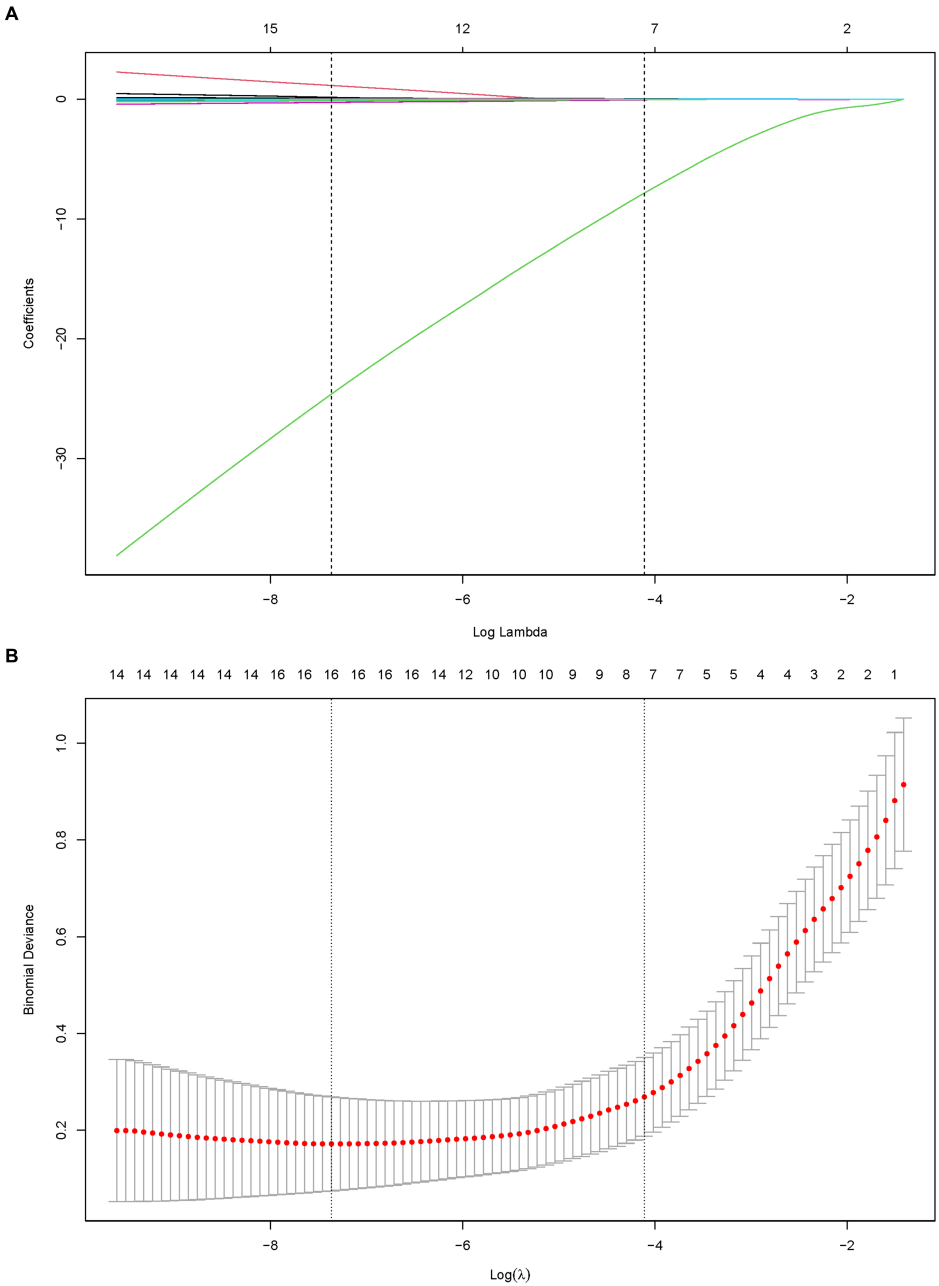
LASSO regression to identify clinical features that may predict unfavorable outcome for TLI patients. **(A)** LASSO coefficient profiles. **(B)** LASSO regression using 10-fold cross-validation and the “minimum plus one standard error” criterion to identify the optimal penalization coefficient lambda (λ). LASSO, Least Absolute Shrinkage and Selection Operator; TLI, Traumatic lung injury.

**Figure 3:**
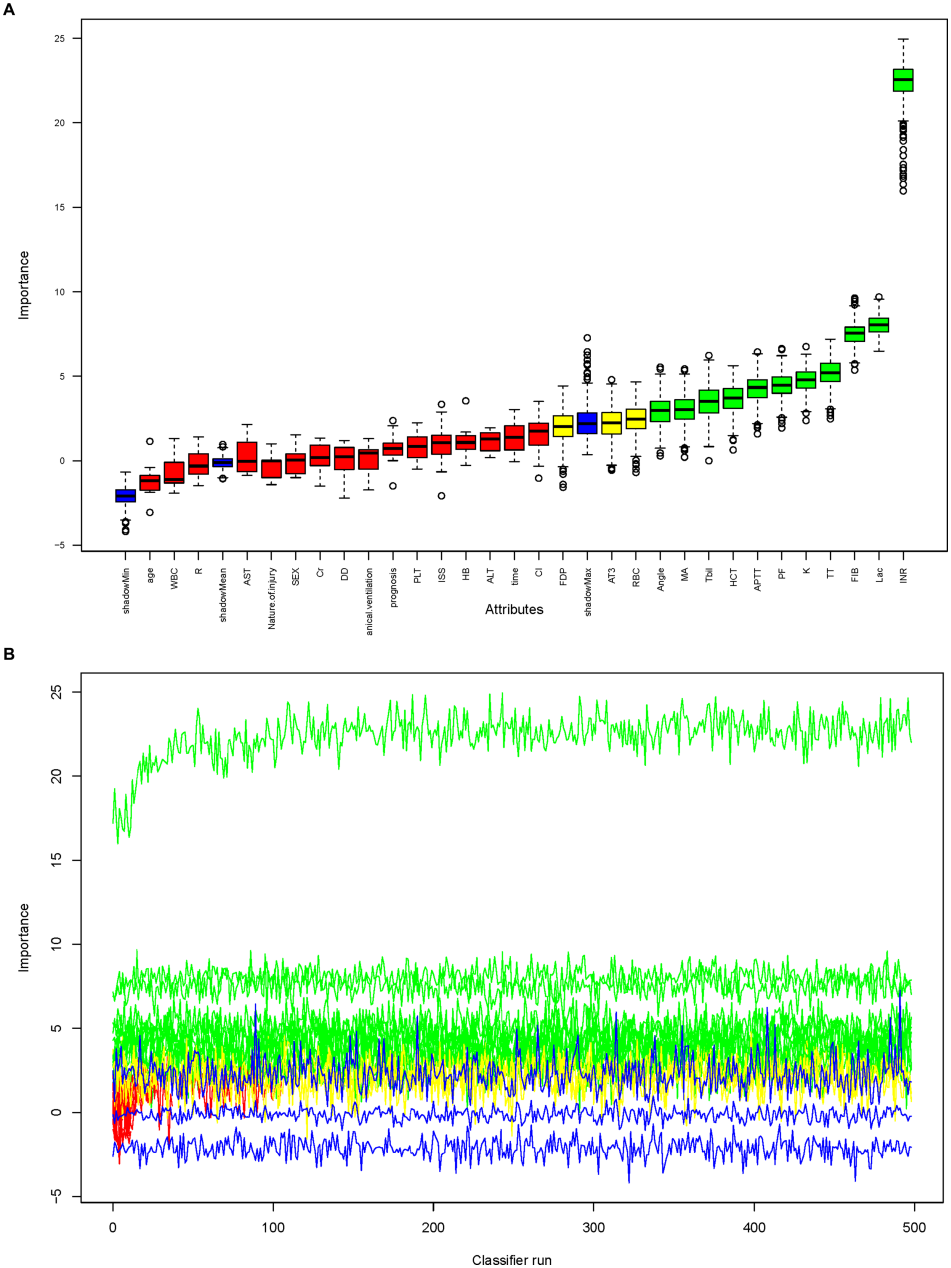

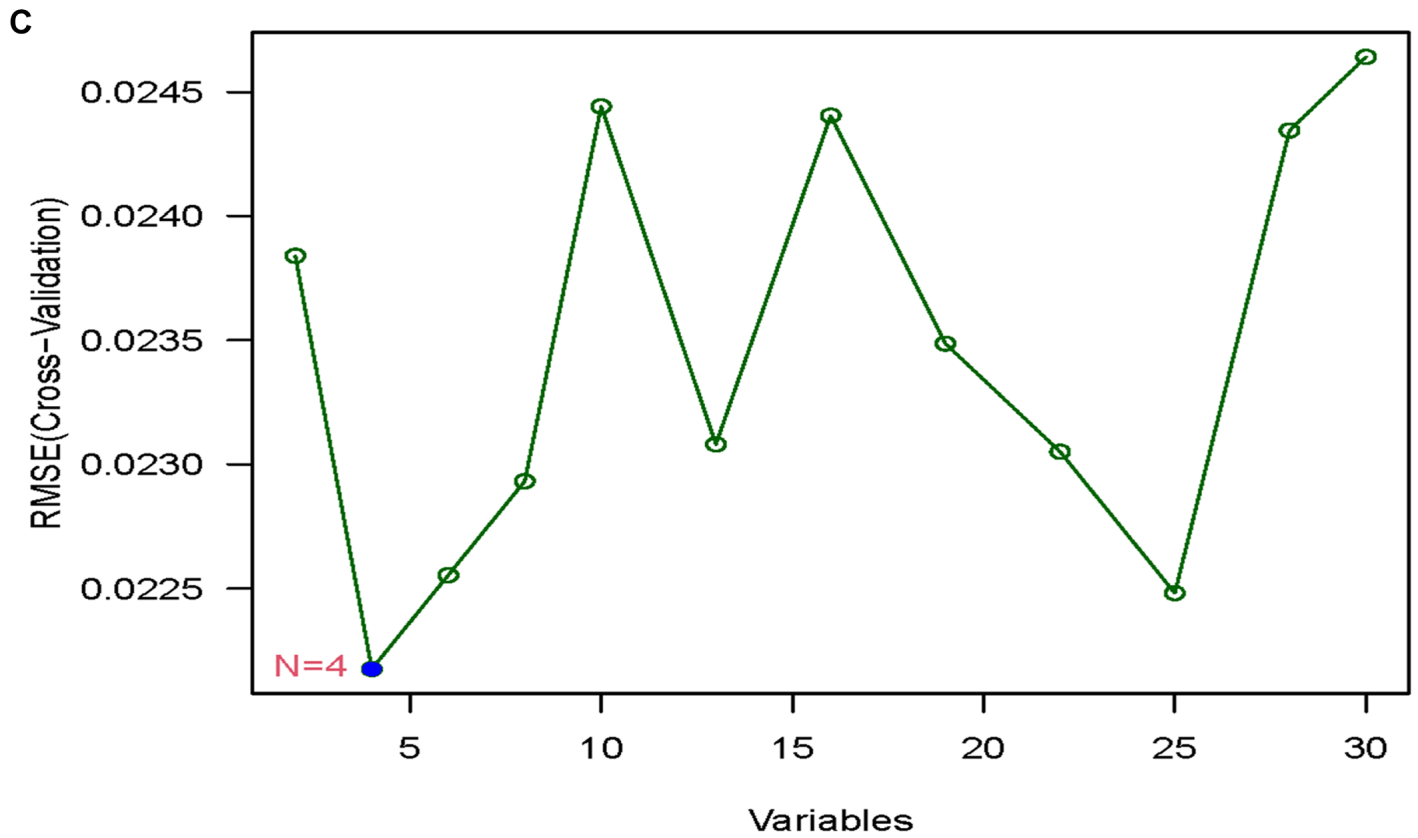
Random forest analysis to identify clinical features that may predict poor prognosis with TLI patients. **(A)** Boxplot for all features in random forest analysis. Green indicates important variables; red, blue, or yellow, rejected variables. **(B)** Rejection or acceptance of factors during random forest classification runs. **(C)** SVM-RFE to identify clinical predictors of poor prognosis with TLI patients. SVM-RFE, Support Vector Machine Recursive Feature Elimination; TLI, Traumatic lung injury.

**Figure 4:**
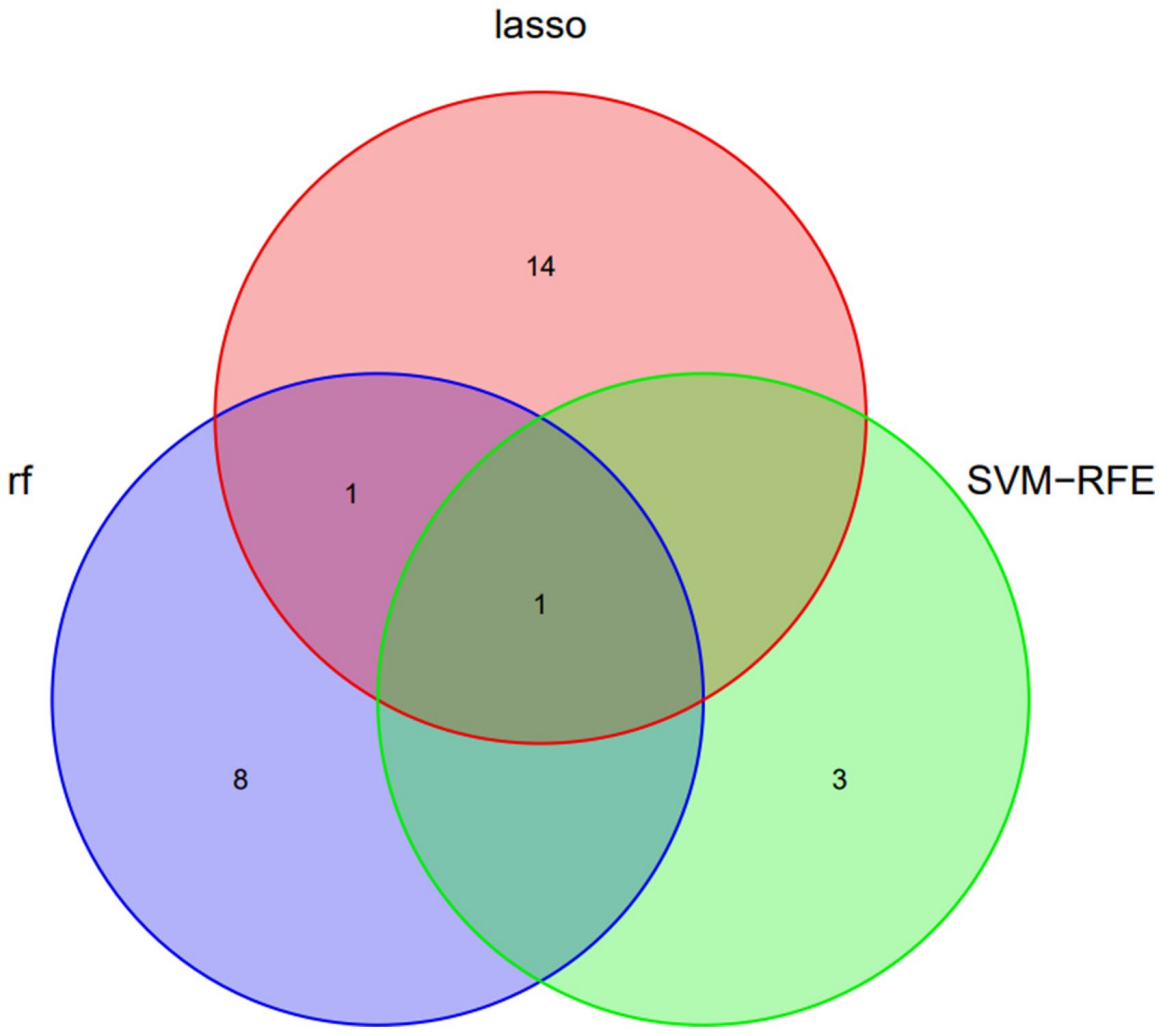
Venn diagram demonstrating the overlap of clinical factors identified by LASSO, Random Forest, and SVM-RFE Methods as predictors of poor prognosis in TLI patients. LASSO, Least Absolute Shrinkage and Selection Operator; SVM-RFE, Support vector machine recursive feature elimination; TLI, Traumatic lung injury.

Three algorithms, LASSO regression, SVM-RFE, and random forest, were utilized to screen for indicators that could predict unfavorable outcome in TLI patients. The LASSO regression analysis identified 16 clinical features: Sex, Age, Mechanical ventilation, INR, Platelet counts, PF, Lactate, ISS, WBC, AST, APTT, Fibrinogen, TT, Antithrombin, D-Dimer, and Length of ICU stay (Figure 2). The random forest analysis identified the same six features as LASSO (INR, lactate, Fibrinogen, TT, PF, and APTT) and the other four features: K, Tbil, MA, and Angle (Figures 3A,B). SVM-RFE identified the same one feature as LASSO and random forest (INR), and the following three features: CI, D-Dimer, and Platelet counts (Figure 3C). All three methods overlap the same factor INR, which is considered a potential predictor of poor prognosis with TLI patients (Figure 4).

### 3.3 Evaluation of INR for predicting unfavorable outcome in TLI patients

**Figure 5:**
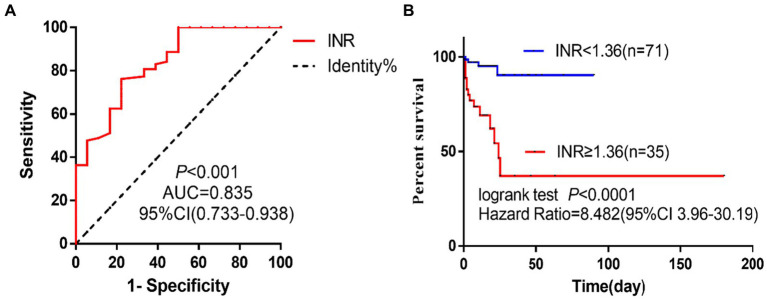
INR value in predicting poor prognosis of TLI patients. **(A)** ROC curve analysis. **(B)** Survival curves. INR, International normalized ratio; TLI, Traumatic lung injury; ROC, Receiver operating characteristic.

Receiver Operating Characteristic curve analysis was performed to evaluate the predictive value of INR for unfavorable outcomes in TLI patients, as shown in Figure 5A. The area under the curve (AUC) was 0.835 (95% CI, 0.733–0.938), indicating a strong predictive power (*p* < 0.0001). The optimal cut-off value for INR was determined to be 1.36, with a sensitivity of 0.778 and a specificity of 0.761. TLI patients were subsequently stratified into two groups based on the INR cut-off value of 1.36: INR ≥ 1.36 and INR < 1.36. The 28-day survival rate of the INR ≥ 1.36 group was significantly higher than that of the INR < 1.36 group (*p* < 0.0001). Furthermore, the 28-day mortality risk of the INR ≥ 1.36 group was found to be 8.5 times higher than that of the INR < 1.36 group (Figure 5B).

## 4 Discussion

This study, for the first time, demonstrates that INR is a risk factor for poor prognosis in TLI patients and proposes that an INR ≥ 1.36 can be employed as an early warning indicator of adverse outcomes in TLI patients. INR is a standardized ratio calculated based on the International Sensitivity Index (ISI) and prothrombin time (PT), which can be used for standardized assessment of hemostasis function (12). PT is the time it takes for blood plasma to clot after the addition of tissue factor and calcium ions. A significant lack of factors VII, X, prothrombin, and fibrinogen in the blood can lead to an extended PT. Among these, factor VII deficiency has the most significant impact (13). During trauma, tissue damage causes a large amount of tissue factor to be released, which can activate factor VII and then promote the formation of a large amount of thrombin through the extrinsic coagulation pathway, resulting in excessive consumption of coagulation substrates (14). Factor VII is often the first coagulation factor to be excessively consumed due to its short half-life of only 6 h (15). Therefore, theoretically, an elevated INR is a sensitive indicator for identifying traumatic coagulopathy (16).

Our study findings reveal that the 28-day mortality risk significantly increases in TLI patients with INR ≥ 1.36. Compared to general trauma patients, TLI patients face multiple unique challenges in coagulation dysfunction: Firstly, TLI patients often experience severe physiological disturbances, such as hypothermia and acidosis, which significantly exacerbate coagulation dysfunction. While these issues may occur in general trauma, they are typically more severe and difficult to correct in TLI patients due to impaired lung function (17, 18). Secondly, TLI patients often suffer from contusions and rib fractures, leading to unique changes in respiratory mechanics. Chest wall instability not only affects respiratory function but may also directly damage vascular endothelium through sustained mechanical stress, activating the coagulation cascade. This mechanism is rare in other types of trauma (19, 20). These factors contribute to hypoxemia, which directly impacts the synthesis and function of coagulation factors and may cause platelet dysfunction and endothelial cell damage. This persistent hypoxic state, uncommon in other trauma types, is a key feature of TLI-related coagulation dysfunction (21, 22). Notably, the inflammatory response in TLI is also unique. Lung tissue damage releases numerous inflammatory mediators that can enter the systemic circulation directly through pulmonary circulation, affecting the body’s coagulation system. This widespread inflammatory response may lead to more severe coagulation imbalances compared to localized trauma (23–28). Thirdly, tissue plasminogen activator (tPA) and urokinase plasminogen activator (uPA), synthesized and released by pulmonary endothelial cells, both facilitate the degradation of fibrin within the blood vessels and exacerbate lung injury. Additionally, uPA promotes the degradation of fibrin in the extrapulmonary matrix, intensifying the edema of pulmonary tissue (29–32). Lastly, TLI patients often require mechanical ventilation support, which can further affect coagulation by increasing intrathoracic pressure and impacting venous return. This treatment-related effect on coagulation is less common in other types of trauma (33). This highlights the importance of early identification and timely correction of coagulopathy in TLI patients.

Interestingly, the diagnostic criteria for coagulopathy resulting from different injury sites are not uniform. The general diagnostic criteria for traumatic coagulopathy are an INR > 1.5 (34); coagulopathy associated with brain injury is diagnosed with an INR > 1.3 (35); our study indicates that an INR ≥ 1.36 is the diagnostic criteria for traumatic lung injury-related coagulopathy. This variation is related to the distinct pathophysiological mechanisms of coagulopathy associated with different injury sites. For instance, brain injury can easily cause intracranial hypertension and neuroendocrine disorders, and the abundant capillaries in the brain are susceptible to endothelial damage, leading to coagulation activation (36). Consequently, the INR value for the diagnosis of brain injury-related coagulopathy is lower than that of lung injury.

Naturally, there are several limitations to this study that should be acknowledged: firstly, the research is a retrospective, single-center analysis with a limited sample size. While the results are suggestive, they require confirmation through multi-center studies to enhance their generalizability and credibility. Secondly, the study population consists of polytrauma patients with traumatic lung injury as the principal injury, rather than patients with isolated traumatic lung injury. This selection is more reflective of the typical clinical scenario. As a result, we meticulously tallied the data from the combined injury sites to ensure that any differences between groups were not attributable to the nature of the injury site. Thirdly, due to considerations of data integrity, this study collected data only at the time of patient admission, without continuous monitoring of coagulation test indicators. Fourthly, hypothermia was not factored into our analysis as a variable. In future research, we are committed to refining and substantiating our experimental findings.

In conclusion, this study has demonstrated that an INR of ≥1.36 can serve as a valuable indicator of poor prognosis in patients with TLI and can be employed as a criterion for initiating treatment for TLI-related coagulopathy.

## Data availability statement

The original contributions presented in the study are included in the article/supplementary material; further inquiries can be directed to the corresponding author.

## Ethics statement

The studies involving humans were approved by the Ethics Committee of the 908th Hospital of Chinese PLA Logistic Support Force. The studies were conducted in accordance with the local legislation and institutional requirements. Written informed consent for participation was not required from the participants or the participants’ legal guardians/next of kin in accordance with the national legislation and institutional requirements.

## Author contributions

QL: Conceptualization, Writing – original draft. EP: Data curation, Writing – review & editing. XD: Data curation, Writing – review & editing. XS: Data curation, Writing – review & editing. LZ: Formal Analysis, Writing – review & editing. LH: Validation, Writing – review & editing. QZ: Validation, Writing – review & editing. JS: Conceptualization, Funding acquisition, Writing – original draft, Writing – review & editing.
